# Designing and using incentives to support recruitment and retention in clinical trials: a scoping review and a checklist for design

**DOI:** 10.1186/s13063-019-3710-z

**Published:** 2019-11-09

**Authors:** Beth Parkinson, Rachel Meacock, Matt Sutton, Eleonora Fichera, Nicola Mills, Gillian W. Shorter, Shaun Treweek, Nicola L Harman, Rebecca C. H. Brown, Katie Gillies, Peter Bower

**Affiliations:** 1 Health Organisation, Policy and Economics (HOPE), University of Manchester, Manchester, UK; 2 Department of Economics, University of Bath, Bath, UK; 3 MRC ConDuCT-II Hub, University of Bristol, Bristol, UK; 4 Institute of Mental Health Sciences, School of Psychology, Ulster University, Coleraine, UK; 5 Health Services Research Unit, University of Aberdeen, Aberdeen, UK; 6 MRC North West Hub for Trials Methodology Research, University of Liverpool, Liverpool, UK; 7 Faculty of Philosophy, University of Oxford, Oxford, UK

**Keywords:** Recruitment, Retention, Trials, Incentives

## Abstract

**Background:**

Recruitment and retention of participants are both critical for the success of trials, yet both remain significant problems. The use of incentives to target participants and trial staff has been proposed as one solution. The effects of incentives are complex and depend upon how they are designed, but these complexities are often overlooked. In this paper, we used a scoping review to ‘map’ the literature, with two aims: to develop a checklist on the design and use of incentives to support recruitment and retention in trials; and to identify key research topics for the future.

**Methods:**

The scoping review drew on the existing economic theory of incentives and a structured review of the literature on the use of incentives in three healthcare settings: trials, pay for performance, and health behaviour change. We identified the design issues that need to be considered when introducing an incentive scheme to improve recruitment and retention in trials. We then reviewed both the theoretical and empirical evidence relating to each of these design issues. We synthesised the findings into a checklist to guide the design of interventions using incentives.

**Results:**

The issues to consider when designing an incentive system were summarised into an eight-question checklist. The checklist covers: the current incentives and barriers operating in the system; who the incentive should be directed towards; what the incentive should be linked to; the form of incentive; the incentive size; the structure of the incentive system; the timing and frequency of incentive payouts; and the potential unintended consequences. We concluded the section on each design aspect by highlighting the gaps in the current evidence base.

**Conclusions:**

Our findings highlight how complex the design of incentive systems can be, and how crucial each design choice is to overall effectiveness. The most appropriate design choice will differ according to context, and we have aimed to provide context-specific advice. Whilst all design issues warrant further research, evidence is most needed on incentives directed at recruiters, optimal incentive size, and testing of different incentive structures, particularly exploring repeat arrangements with recruiters.

## Background

Randomised controlled trials (RCTs) used to determine the efficacy and effectiveness of new healthcare interventions depend on successful recruitment and retention of trial participants. Nevertheless, approximately 45% of trials fail to recruit the necessary number of participants in the time planned [1], a figure that has changed little over time [2, 3]. Despite their importance, very little evidence exists on effective methods to boost recruitment and retention [4, 5].

The use of incentives in trials has been proposed as a strategy to improve recruitment and retention [6]. An incentive is generally defined as anything ‘that motivates or encourages someone to do something’ [7], although the use of the term in the context of trials tends to be narrower. Whilst incentives are often financial, they can take many forms in the trial context. The effects of incentives are complex and depend on how they are designed, the form in which they are given, how they interact with other motivations, and what happens after they are withdrawn [8].

The aim of this paper is to use a scoping review to ‘map’ the literature, with two aims: to develop a checklist on the design and use of incentives to support recruitment and retention in trials; and to identify key research topics in this area for the future.

## Methods

We conducted a ‘scoping review’, which is an appropriate methodology for ‘mapping the field’ in terms of the existing evidence around incentives in trials, and for providing initial guidance to assist decision-making about how incentives might be used to support recruitment and retention in trials [9, 10]. We reported the study according to the new guidelines for scoping reviews [11]. There was no review protocol.

We sought to identify literature relevant for informing the design and implementation of incentive schemes in trials. We drew on theoretical literature about incentives and the issues in their design that are important, as well as empirical literature examining these design issues in practice. Theoretical and empirical literature was identified using our prior knowledge of the seminal works in this area, and additional empirical literature was identified through a structured search of PubMed and EconLit (the search strategies are provided in the Appendix). As the literature on the use of incentives in trials is limited, we drew upon evidence from two other healthcare settings in which incentives are commonly used: pay for performance, and health behaviour change. The ORCCA database was launched in September 2016, bringing together published studies and ‘work in progress’ on recruitment [12]. We updated our search in September 2018 by assessing studies relating to ‘incentives’ in the ORCCA database.

The focus of this review was on issues relating to the design of incentives, aimed at both participants who are being recruited or retained and those doing the recruitment and retention. When drawing on literature examining the use of incentives in other healthcare settings, the evidence on pay for performance in healthcare is likely to be most informative in terms of recruiter incentives as pay-for-performance incentives tend to target providers rather than patients. The evidence on the use of incentives for health behaviour change, on the other hand, largely examines patient-directed incentives, and so is likely to be most relevant to incentives aimed at participants who are being recruited or retained.

Although we highlight some ethical issues, a detailed consideration of the ethical issues surrounding incentives was beyond the scope of this paper. An overview of the issues can be found in the NHS Health Research Authority guidance on payments and incentives in research [13].

We first examined the identified papers, searching for the key design issues which were evident in the theoretical or empirical literature known to the authors or identified in the search. The design issues which emerged from this initial examination were discussed among the study team and eight key design issues were agreed upon.

Once this list of design issues was agreed, we sought to review the evidence pertaining to each. For each design issue, we first examined the literature from a trials setting, starting with systematic reviews. If there were no systematic reviews specific to the design aspect in the trials, or the systematic reviews from a trials setting found insufficient evidence to draw conclusions, we then examined single studies from a trials setting, alongside evidence from the two other settings (pay for performance and health behaviour change). Within the evidence on pay for performance and health behaviour change, we adopted the same approach of using systematic reviews, and then single studies when reviews were not available. Laboratory or field experiments and solely qualitative studies were excluded. As with many scoping studies, we did not assess the quality of the individual reviews and studies [9].

The results are presented as issues to be considered when designing an incentive scheme to improve recruitment and retention in trials. For each issue, the relevant economic theory is presented, followed by a summary of the empirical evidence. This was then synthesised in general guidance around incentive design, reflecting on whether the theoretical predictions appear to be borne out in practice. These recommendations are summarised in a checklist to help design incentive schemes. Although we did not formally adopt the approach, our analytical approach is in line with the realist approach, moving away from specific statements about ‘what works’ in favour of ‘contextual advice in the general format: in circumstances such as A, try B, or when implementing C, watch out for D’ [14].

## Results

**Fig. 1:**
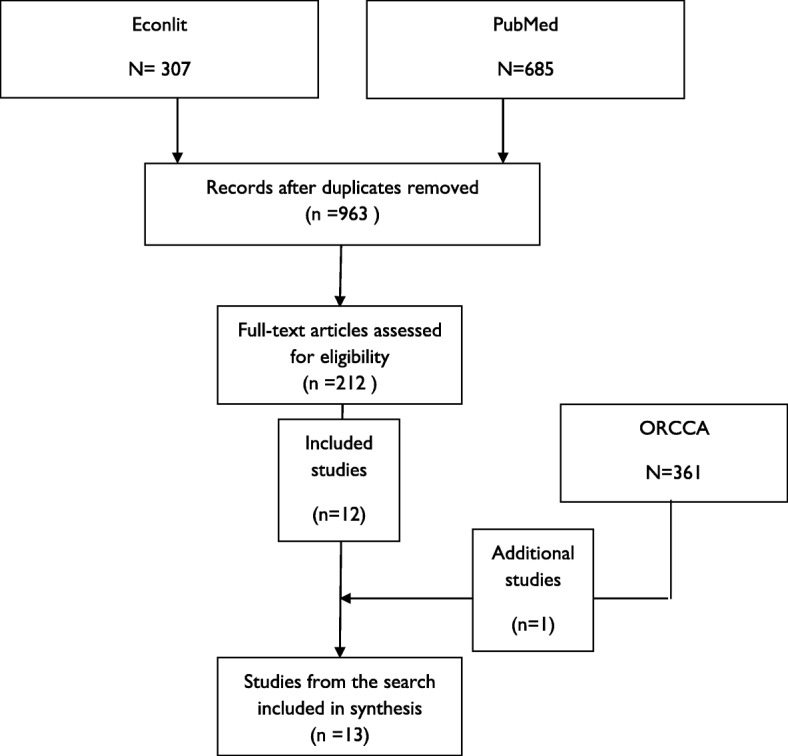
Modified Preferred Reporting Items for Systematic Reviews and Meta-Analyses (PRISMA) diagram for scoping review. ORCCA, Online Resource for Recruitment Research in Clinical Trials

The structured search identified 307 articles from EconLit and 685 articles from PubMed, presenting 963 unique records after duplicates were removed, and we assessed 212 full-text articles for eligibility, of which 12 were included in the review (additional to those already known to the authors). The search on ORCCA identified 361 studies, of which one additional study was included in the review (see Fig. 1 for a PRISMA diagram modified for the scoping review, and Additional file 1 identifies which papers came from each search).

### The theory of incentives

Economic theory would characterise the relationships between the investigator, recruiters, and trial participants as ‘contracts’ between a principal and multiple agents [15]. In this relationship, the investigator (the principal) contracts with recruiters (group 1 agents) to recruit and retain participants (group 2 agents) into trials. Recruiters incur time and financial costs associated with recruiting and retaining participants in the trial. Participants may incur direct costs such as travel expenses, and opportunity costs in terms of time that could have been spent on other activities. The problem for the investigator is to boost both recruitment and retention, whilst obtaining a representative pool of informed and engaged participants who will complete the trial.

Currently, recruiters may be incentivised on the number of potential participants screened for eligibility or recruited, not on their actual eligibility or other key factors such as the quality of data collection and record-keeping about recruitment processes. Participants may not be directly incentivised at all.

Incentive theory states that the key is to align the parties’ interests so that all agents (both those recruiting and those participating) will choose the optimal effort level that brings about the desired recruitment and retention rates [15]. This can be achieved by tying the benefit of the agents to that of the investigator, usually by setting incentives that are linked to variations in some measure of recruitment and retention rates and the appropriateness of participants.

### Design issues to consider

In the following we present the eight issues to consider when designing an incentive scheme to improve recruitment and retention. The relevant theoretical and empirical evidence is summarised and recommendations are made based on this. These are also summarised in Table 1 as a checklist for incentive scheme designers to follow.

**Table 1:** Checklist for incentive scheme design

Design aspect to consider	Evidence	Issues to consider	Future research priorities
1. What are the current incentives and barriers operating in the system?	• Must complement the existing incentives already operating in the setting, and work to overcome the current barriers • Consideration of the current state of play in a field is a key step but is frequently overlooked and can be affected by availability of information	• What incentives are in the setting already? • What are the main barriers to recruitment?	• Understanding current systems of incentives operating in trials
2. Who should incentives be directed towards?	• Choice should depend on where the greatest barriers exist, where accountability for improvement lies, and where the greatest gains may be achieved for the available resource • Whilst there is more evidence to support patient incentives, all options show some promise	• Where do the greatest barriers to recruitment/retention currently lie? With participants, recruiters, or both? • If the barriers are with recruiters, do individuals have the ability to overcome these, or is a team effort required?	• Testing organisational and individual incentives for recruiters, and shared incentive schemes
3. What should be incentivised?	• Incentives linked to processes generally found to be more effective than outcome-linked incentives, although this evidence is from settings other than trial recruitment and retention • There must be evidence of a strong causal relationship between the incentivised process and the desired outcome if process-based incentives are to achieve the overall aim	• What is the desired outcome? Recruitment, retention, or both? • Would linking incentives directly to this outcome transfer unfair risk onto participants or recruiters? • What processes may lead to this outcome? Is there evidence of a strong causal relationship between processes and this outcome? • What other outcomes are important? Will these be neglected if not incentivised?	• Testing the relative benefits of process and outcome incentives and of incentivising a single metric compared to a range of measures
4. What form of incentive should be offered?	• The psychological effects of monetary incentives do not appear to crowd out the direct price effect • Monetary incentives were found to be more effective than non-monetary incentives for participants	• Who is the incentive directed towards? What are they likely to value most or be motivated by? • Is it possible to provide monetary incentives? • What is the overall budget for incentive provision?	• Testing of the relative effectiveness of monetary compared to non-monetary incentives for recruiters
5. How large should the incentive be?	• Larger incentives should be more effective • Size of incentive needed will be very context dependent, increasing in situations that require more effort from participants or recruiters, or more risk • Incentive size will determine the overall cost of the scheme	• How are agents currently reimbursed? • How much effort is required from participants or recruiters? • How large is the risk associated with trial involvement? • What is the overall budget for incentive provision? • Would an incentive of the chosen size raise concerns around coercion?	• Testing the cost-effectiveness of larger incentives, accounting for the overall impact on study timelines and costs
6. How should the inventive be structured?	• Incentive structure is crucial in determining the total cost of the scheme • Most effective structure will vary by the context, and the evidence in this area is sparse • Evidence suggests there is no difference in effectiveness between guaranteed and lottery-based incentives for patient incentives • Repeat arrangements with recruiters may warrant exploration of more complex incentive structures	• Who is the incentive directed towards? • If directed towards recruiters, is this a one-off situation or are repeat arrangements likely? • What is the overall budget for incentive provision? • Is budget certainty required from the outset? • Do agents face different barriers to recruitment and retention?	• Exploration of the effects of more complex incentive structures
7. When, and how often, should payments be made?	• Immediate incentives are generally found to be more effective than those paid out in the future • The time between the occurrence of the desired behaviour and the incentive should be minimised	• When can incentives be practically provided in the trial? • Is it possible to provide multiple incentives over time?	• Testing the benefits of multiple incentives over time
8. What are the potential unintended consequences?	• In addition to their impact on recruitment and retention, the introduction of incentives may also result in unintended consequences • Incentives should be designed to minimise the opportunities for individuals to engage in undesirable behaviours, and potential unintended consequences should be monitored	• Is incentive provision likely to lead to undue inducement or coercion of participants? • Can exclusion criteria be easily verified? • Are recruiters likely to game the system? • What impacts are incentives likely to have in the long run? • How can opportunities for individuals to engage in undesirable behaviours be minimised? • What monitoring could be put in place to ensure quality trial conduct?	• Evaluating the extent to which potential unintended consequences materialise in practice

### What are the current incentives and barriers operating in the system?

The first design issue to consider differs from the others, in that it does not derive from a specific theory or relate to particular empirical findings. Rather, here we highlight the need to understand the current context into which new incentive mechanisms are to be introduced.

When designing an incentive system, it is vital to consider the existing incentives already operating in trials, and the current barriers to recruitment and retention. For participants, the potential to access new treatments and altruistic benefits to wider society may act as incentives to participate in trials. Participants in a system of care free at the point of use may have different existing incentives to those who face co-payments.

Participants may experience barriers to trial participation including additional demands such as attending appointments and associated time, effort, or financial costs, discomfort associated with trial procedures, the risk of not being allocated to their preferred treatment, and uncertain outcomes [16].

Recruiters to trials may be researchers, specialist recruiters, or clinicians. For specialist recruiters, their income may be linked to trial recruitment. All may be incentivised by the potential for improved care for participants, altruism, career advancement, co-authorship of scientific outputs, and the opportunity to keep up to date with current research. Recruiters may face time constraints and a lack of resources, with clinicians acting as recruiters facing additional concerns over potential threats to the doctor–patient relationship and a loss of professional autonomy [16].

#### Conclusion

All institutional arrangements create incentives, even if they are not explicitly labelled as such. Consideration of the incentives created by the current context is a key step frequently overlooked in the design of incentive schemes. The most effective incentives are those that address existing barriers. Incentives will have a muted effect if they conflict with existing incentives already operating within the system. Transparency on current systems of payments and incentives would be helpful to inform further research into what works and in what setting.

### Who should incentives be directed towards?

Incentives could be directed to participants, recruiters (individuals, teams or sites), or a combination.

#### Incentivising participants

##### Theory

Individuals are motivated by actions that produce measurable and tangible benefits [17]. Many factors working against trial participation are tangible (such as time and travel costs), while benefits (such as health improvements, access to new treatments, or the wider benefits of research) are often uncertain or occur far in the future. In the context of screening or prevention, economic theory suggests the use of subsidies or financial incentives to correct for suboptimal health choices [18, 19]. Similarly, offering incentives to participants can provide an immediate tangible benefit which may offset some barriers.

##### Evidence

A Cochrane systematic review of strategies to improve retention in randomised trials found that provision of a monetary incentive was effective (relative risk (RR) 1.18; 95% confidence interval (CI) 1.09 to 1.28) [4]. Of the six strategies tested in the included studies, monetary incentives demonstrated the clearest impact on retention. However, the majority of the included studies evaluated rates of questionnaire responses rather than strategies to improve retention rates when participants are required to return to sites for follow-up assessments. The 2018 update of the Cochrane review on recruitment identified two studies and concluded that incentives probably improve recruitment (risk difference = 4%; 95% CI = − 1% to 8%) [5]. This update included a study where the financial incentive was conditional upon attending a screening visit.

#### Incentivising recruiters

##### Theory

Theory suggests that directly linking payment to the individual responsible for improvement provides stronger motivation than linking to groups (e.g. a whole recruitment site) [20]. However, there may be a trade-off between the power of incentives and the reliability of performance monitoring when applied to individuals [21]. When targeting groups, members may hope to benefit from increased efforts from others rather than increasing their own effort – so-called *free riding* [22]. Peer monitoring, and cooperation can reduce free-riding [23]. Additionally, if barriers to recruitment or retention are due to system failures, this is where incentives should focus [24].

##### Evidence

A systematic review of the effectiveness of payment to healthcare professionals for recruitment of participants to trials identified three relevant studies, concluding that the evidence was very limited, of poor quality, and inconclusive [25].

Reviews of the evidence from pay for performance in healthcare suggest that both organisational and individual incentives can produce significant improvements in activity [26], with larger effects generally found when targeting smaller units (individuals/teams vs organisations) [27].

#### Incentives targeting both recruiters and participants in the same study

Only one trial was identified that directly compared the effectiveness of provider, patient, and shared incentives. Whilst the outcome of interest was treatment adherence rather than recruitment and retention, shared financial incentives were found to be effective whereas incentives to physicians or participants alone were not [28]. A systematic review of pay for performance found that whilst combined incentives were rarely used, they did lead to positive results in the two studies identified [27].

##### Conclusion

The choice of who to incentivise should depend on where the greatest barriers exist, where accountability for improvement lies, and where the greatest gains may be achieved. Whilst there is more evidence to support participant incentives, all options show some promise. The testing of organisational and individual incentives for recruiters, and shared incentive schemes between recruiters and participants, should be encouraged.

### What should be incentivised?

Incentives could be linked to:

- Processes that may lead to increased recruitment and retention (e.g. number of participants invited, reminders sent)
- Outcomes (number of participants successfully recruited or retained)
- A combination of processes and outcomes

#### Theory

In the simplest principal–agent framework, where agents’ efforts and performance are perfectly observable, incentives are linked to the desired outcome(s). However, outcomes are rarely determined solely by the actions of agents, and so become a ‘noisy’ signal of actual effort [29]. Outcome-based incentives transfer risk to the agent and may be inequitable, for example if some recruiters are dealing with more complex populations and must consequently work harder to recruit or retain each additional participant. Linking incentives to process indicators may therefore be more effective in inducing effort as these are under the direct control of the agent [26].

However, there must be evidence of a strong causal relationship between the incentivised process and the desired outcome if process incentives are to achieve the overall aim of increasing recruitment and retention. There is a danger that increases in process measures may not translate into increased recruitment or retention, or may lower the overall quality of the participant pool.

The role of agents is likely to comprise multiple tasks, only some of which the investigator can verify and therefore link to incentives. This multi-tasking problem can lead to concerns that attaching a large incentive to only one task or measure may lead to effort diversion away from other non-incentivised tasks [30], such as recruitment over retention. This is the case if tasks are substitutes, for example if they are both time-consuming but unrelated. Alternatively, tasks may be complements, meaning that improvements in one area can lead to wider improvements in other areas [31]. Using a broad array of performance measures (including a mix of process and outcome metrics) minimises the risk of effort diversion, but increases the complexity and resources required to implement the incentive scheme [32].

#### Evidence

No studies examining the effectiveness of process versus outcome-based incentives for trial recruitment or retention were identified, but this issue has been examined in the literature on pay for performance in healthcare. Two systematic reviews (one including a meta-analysis) concluded that incentives linked to process indicators generally yielded greater quality improvements than incentives linked to outcomes [27, 33].

The evidence from the literature on incentives to promote health behaviour change is weaker and more mixed. A Cochrane systematic review of incentives for smoking cessation found that in four trials specifically targeting pregnant women, incentives linked to successful quit attempts (outcome-contingent incentives) resulted in higher quit rates than fixed payments for attending antenatal appointments (non-contingent process incentives) [34]. Conversely, a systematic review and meta-analysis of incentives for weight loss amongst obese populations found a weak although non-statistically significant trend in favour of incentives linked to behaviour change (process) rather than weight loss (outcome) [35].

#### Conclusion

Incentives linked to processes have generally been found to be more effective than those linked to outcomes, although this evidence is from settings other than trial recruitment and retention. There must be evidence of a strong causal relationship between the incentivised process and the desired outcome if process-based incentives are to achieve the overall aim of increasing recruitment or retention. Testing the relative benefits of process and outcome incentives and of incentivising a single metric compared to a range of measures would be informative.

### What form of incentive should be offered?

Earlier, we provided a very broad definition of incentives as anything ‘that motivates or encourages someone to do something’ [7]. In the context of trials, this might involve different categories, including:

- Reimbursement for actual expenses incurred (e.g. payment for a patient to travel to research visits, screening)
- Reimbursement for opportunity costs (e.g. payment for professional time spent discussing a study with a prospective patient)
- Additional incentives to encourage desired behaviour (e.g. prize lottery for completion of research assessments)

Incentives can also take many forms, including:

- Cash or cash-like rewards (money, vouchers, reimbursement for expenses/time/uncomfortable procedures, additional resources for recruiters, etc.)
- Social, emotional, or tokenistic rewards (gifts, donation to charity)
- Reputational incentives, praise and social recognition (such as authorship on research papers), and public reporting

#### Theory

Incentives have two effects; the direct price effect which makes the incentivised behaviour more attractive, and an indirect psychological effect [8]. There are concerns that the psychological effect may work in the opposite direction to the price effect, crowding out the incentivised behaviour. The provision of incentives can have an indirect psychological effect by altering an individual’s perception of the desired behaviour, for example leading them to infer that it may be difficult or unpleasant [36]. Incentives may also signal a market relationship, changing an individual’s decision frame from social to monetary, potentially crowding out their intrinsic motivation [23]. It is unknown which effect will dominate, and this may be context dependent. Providing rewards of a social nature may limit the extent to which incentive provision crowds out intrinsic motivation. Rewards viewed as a ‘splurge’ (such as a computer tablet) that an individual would not normally buy for themselves can be perceived as more valuable than the equivalent cash amount [32].

#### Evidence

A Cochrane systematic review and meta-analysis of strategies to improve retention in randomised trials found that whilst monetary incentives significantly increased the response rate to postal (RR 1.18; 95% CI 1.09 to 1.28) and electronic (RR 1.25; 95% CI 1.14 to 1.38) questionnaires, there was no evidence that offering non-monetary incentives increased retention compared to no incentive (RR 0.99; 95% CI 0.95 to 1.03) [4]. There was little evidence concerning incentives to improve participant return to sites for follow-up.

Another Cochrane systematic review and meta-analysis of methods to increase responses to postal and electronic questionnaires in epidemiological studies identified 13 trials specifically comparing the effectiveness of monetary and non-monetary incentives [37]. Whilst this study did find that non-monetary incentives were effective compared to no incentive, the odds of response were almost doubled when using monetary incentives as opposed to non-monetary incentives (OR 1.87; 95% CI 1.73 to 2.04). Similarly, a meta-analysis of the effectiveness of incentives on electronic health survey response found, by indirect comparison, that monetary incentives had a stronger impact on response than non-monetary incentives compared to a no-incentive comparison (OR 2.43 vs OR 1.33) [38].

#### Conclusion

The psychological effects of monetary incentives do not appear to crowd out the direct price effect, at least when incentives are directed at participants, with monetary incentives found to be more effective than non-monetary incentives. Testing of the relative effectiveness of monetary compared to non-monetary incentives for recruiters is needed.

### How large should the incentive be?

#### Theory

Theory suggests that performance will be positively related to incentive size. However, the marginal increases in performance are expected to diminish as incentive size grows, because of diminishing marginal utility of income and because every unit of performance improvement becomes harder to obtain than the last [39]. The incentive recipient must be compensated for the incremental net costs of undertaking the desired action [15].

When individuals are not fully in control of the relevant outcomes, they require larger incentives to offset the risk of failure [22]. This may mean that trials involving new treatments (where the outcomes for participants are more uncertain) could require larger incentives. However, incentives which are too large may cause ethical challenges such as coercion, and could impair intrinsic motivation.

The relative importance of the incentive in relation to other sources of income will also determine its effectiveness in motivating agents [40]. It is therefore important to consider how participants and recruiters are currently reimbursed. A theoretical model for price setting in pay-for-performance schemes shows that optimal prices should reflect the marginal benefit to the payer of the outcomes achieved, providers’ altruism, and the opportunity cost of public funds [41]. This framework could be adapted for use in trials.

The size of incentives used in the literature is often relatively modest [38], with a recent payment to UK patients of £100 [42]. Larger incentives could raise issues around coercion, which are discussed in the NHS Health Research Authority guidance [13], although the levels at which an incentive becomes coercive is likely to depend on the context, including the population, the burden associated with the trial, and the recruitment and retention incentives.

#### Evidence

Two Cochrane systematic reviews and meta-analyses found that higher-value incentives significantly increased the odds of response to postal questionnaires [4, 37]. However, responses may differ when participants are required to attend in person.

Two systematic reviews and meta-analyses of the literature on health behaviour change concluded that there was no evidence that larger incentives were associated with greater behaviour change [43, 44].

The results from studies of pay for performance in health care are mixed. Whilst four systematic reviews found no clear relationship between incentive size and performance [26, 27, 39, 45], a more recent systematic review and meta-analysis estimated that the odds of showing a positive effect were three times higher for schemes with larger incentives [33].

#### Conclusion

In theory, larger incentives should be more effective. However, the size of incentive needed will be very context dependent, increasing in situations that require more effort from participants and recruiters or involve more risk. Ethical issues around the size of the incentive require consideration; care should be taken that individuals are not coerced into participation due to their personal circumstances, and a large incentive may signal risk. The size of the incentive will determine the overall cost of the scheme, and may therefore need to be restricted. There is a need to provide evidence on the cost-effectiveness of larger incentives, accounting for the overall impact on study timelines and costs.

### How should the incentive be structured?

Incentives are commonly thought of in relatively simple terms, with a set amount of money linked to a given task. However, there are many possible ways in which to structure incentive systems, including:

1. Guaranteed payments versus lotteries
2. Bonuses versus penalties
  - Bonuses – additional payments for performance
  - Penalties – payments withheld for below-target performance
  - Deposit contracts – a hybrid where individuals deposit bonuses which are only returned if they meet their targets, and are forfeited if they fail
3. Absolute versus relative reward structure
  - Absolute – payment for achieving a pre-defined level of performance; all agents can potentially receive the incentive
  - Relative – a tournament where a percentage of the top performers receive the incentive; agents compete
4. Rewards for achievement levels versus improvements in achievement
5. Graduated or tiered bonuses with incentives triggered at multiple levels of performance
6. Shared saving programme: savings to the investigators resulting from reduced trial length or attrition rates could be shared with recruiters

Whilst alternative structures such as lotteries could be applied to both participants and recruiters, some design options (such as relative reward structures) are only applicable to recruiters as these involve performance evaluation across agents.

#### Theory

Along with incentive size, the incentive structure is crucial in determining the total cost of the scheme. Lotteries or tournaments provide budgetary certainty to the investigator as a fixed amount will be paid out, and can reduce overall costs as not all agents will receive incentive payments. Relative performance evaluation across agents can also filter out common risks (such as a small number of eligible participants for certain treatments) which may affect the absolute level of performance achieved [46]. However, relative thresholds generate uncertainty which can deter effort, since the level of performance necessary to gain the reward in a tournament is unknown [32]. When agents face different barriers to recruitment and retention (such as varying eligible populations), or are considered to be risk averse, absolute thresholds may be more effective [29].

Motivation depends on baseline performance, with thresholds that are perceived as unachievable unlikely to induce effort [47]. Conversely, if baseline performance already exceeds the threshold, there is no incentive for improvement. High fixed targets or tournaments based on absolute performance will tend to reward current high achievers, rather than induce additional effort from low achievers [20]. A series of tiered thresholds or incentives based on improvement in performance may therefore be more effective in inducing continuous effort than one absolute threshold [32]. However, increasing the complexity of an incentive scheme can weaken the behavioural response as it becomes more difficult for agents to compute the likely relationship between effort and reward [32].

Theory suggests that penalties should generate larger impacts than bonuses as individuals are more sensitive to losses [48]. However, penalties could put further strain on under-resourced agents, and it may be difficult to persuade agents to opt in to such schemes, or they may opt out as soon as they experience losses.

#### Evidence

A Cochrane systematic review and meta-analysis concluded that there was no clear evidence that guaranteed monetary incentives were more effective than prize draws for improving postal questionnaire response rates, but this was based on just two studies [4].

A systematic review of systematic reviews of pay for performance found that studies tend to find more positive effects when absolute rather than relative targets are used, with results suggesting that multiple tiered targets may contribute to positive effects [39]. For example, the Quality and Outcomes Framework (QOF) has a minimum performance threshold below which no payment is made, a maximum threshold above which no additional payments are made, and a linear payment schedule in between [49]. The same review concluded that there was very little evidence on the relative effectiveness of bonuses compared to penalties [39]. Just one relevant study was identified, which found some evidence of increased effectiveness for programmes based on ‘new money’ (bonuses) compared to those relying on reallocation of existing funds (effectively penalties) [27].

A systematic review and meta-analysis of patient incentives for changing health behaviours found that the effect of financial incentives was not modified by attainment certainty (guaranteed payments versus lotteries) [44]. A Cochrane systematic review concluded that comparisons between reward-based and deposit-refund interventions need further investigation as the current evidence is lacking [34].

#### Conclusion

Incentive structure is crucial in determining the total cost of the scheme. The evidence in this area is sparse, but the most effective structure will likely vary by context. For patient-directed incentives at least, the evidence suggests there is no difference in effectiveness between guaranteed and lottery-based incentives. Nevertheless, these conclusions are based upon a limited number of studies and so further research would be informative. Repeat arrangements with recruiters may warrant exploration of more complex incentive structures, and tests of different models should be a priority for future research.

### When, and how often, should payments be made?

Payments can be a one-off or split into multiple payments over time.

#### Theory

Behavioural economics suggests that a series of small incentives may be more psychologically motivating than a single payment of the equivalent value [50]. Similarly, reducing the time between the occurrence of the desired behaviour and receipt of the linked incentive is also theorised to increase the behavioural response, as individuals place greater value on things occurring in the present than in the future. For example, payments to patients for clinic visits paid out at those visits should be more effective than withholding the payments until the end of the trial.

#### Evidence

Two systematic reviews concluded that upfront incentives were significantly more effective than the promise of the same incentive in the future in recruiting both participants [37] and general practitioners [51] to participate in research surveys. This finding was also confirmed in a Cochrane systematic review of incentives to improve adherence to tuberculosis treatment [52]. A systematic review of pay-for-performance programmes also provided some weak evidence that the timing of incentives was related to effectiveness, finding that programmes without a delay in incentive payouts were all relatively successful [53].

#### Conclusion

The timing of incentive receipt is important, with immediate incentives generally found to be more effective than those paid out in the future. The time between the occurrence of the desired behaviour and incentive payout should be minimised.

### What are the potential unintended consequences?

The final aspect to consider is the potential consequences of the chosen incentive. In addition to the intended increases in recruitment and retention, the use of incentives has the potential to induce a number of unintended consequences.

#### Ethical implications

Incentives may alter a participant’s decision-making process, potentially resulting in failure to appropriately make an informed choice about the risks and benefits of participation and the balance between the two. Whilst incentives are designed to alter the decision frame, ethical issues may be raised if incentives are deemed to go beyond motivation or encouragement, crossing the line to coercion [13]. Financial incentives may raise ethical issues if they lead to undue inducement, particularly amongst participants who have lower incomes [54, 55].

#### Changes to patient composition and behaviour within the trial

Incentives may induce different types of participants in terms of both observable (e.g. income, age, illness severity, etc.) and unobservable (e.g. level of altruism) characteristics. Whilst provision of incentives could improve the generalisability of trial results if they attract a more representative sample [42], they could also have adverse effects. Care must be taken to keep monitoring trial quality, as the provision of incentives is no guarantee that activities will be conducted per protocol.

When exclusion criteria cannot easily be verified, financial incentives may cause participants to conceal information [56]. Participants may also feel pressure to report improved outcomes or neglect to tell researchers about negative outcomes because they are being paid. Not only could these potential unintended behaviours affect the validity of trial outcomes, they could also put participants and subsequent patients at risk.

#### Gaming

Rather than respond to the incentives by improving effort and performance, agents may simply make their performance appear better through manipulation of the reporting systems used to measure performance [57, 58]. This issue may be accentuated when recruiters are paid by processes rather than outcomes, since they are generally self-reported and more easily manipulated [59]. For example, if the incentive scheme was tied to recruitment processes such as invitations, recruiters may over-report the number of participants they have invited.

#### Legacy effects

Monetary incentives may change how tasks are perceived by agents, weakening intrinsic motivation. As a result, incentives may therefore be effective in the short run but be counterproductive in the long run, causing agents to pursue the desired outcomes less eagerly once the incentive is removed than they would have done before it was introduced [8]. Providing incentives at recruitment only could therefore have detrimental effects on retention. This may also result in legacy effects, where the provision of incentives becomes expected by participants and recruiters. The provision of incentives in one trial could therefore have detrimental effects on effort levels for future non-incentivised trials. Alternatively, incentivised activities can become ingrained in routine behaviour and continue after the incentive is removed, making future incentives superfluous.

#### Conclusion

In addition to the intended impacts, introducing incentives for recruitment and retention has the potential to induce unintended consequences which may affect trial validity and outcomes. Incentives should be designed in such a way as to minimise the opportunities for individuals to engage in undesirable behaviours, and potential unintended consequences should be identified early as part of the trial design process. Along with evaluating the effectiveness of incentives, future research should also investigate the extent to which potential unintended consequences materialise in practice. Incentives should be seen as a tool; other methodological processes should be carefully monitored to ensure quality trial conduct.

## Discussion

### Main findings

Recruitment and retention of participants is critical for trial success, yet both remain significant problems. This paper aimed to provide guidance on the design and use of incentives for participants and staff to improve recruitment and retention in trials. Evidence both in terms of the economic theory of incentives and the empirical literature examining the use of incentives in healthcare has been summarised, to offer guidance for those considering the use of incentives to improve trial recruitment and retention.

The issues to consider when designing an incentive system are summarised into an eight-question checklist for trialists to use. These questions cover: the current incentives and barriers operating in the system; who the incentive should be directed towards; what it should be linked to; the form of incentive; the incentive size; the structure of the incentive system; the timing and frequency of incentive payout; and consideration of the potential unintended consequences.

The evidence demonstrates that the design of incentive systems can be very complex. Specific detail is often overlooked, and all decisions may have both desired and undesired consequences. Whilst not always effective, the evidence shows that incentives can increase effort, but how schemes are designed is a key determinant of their effectiveness, and what works best is context specific. Our guidance is designed to help to make these decision-making processes more rigorous and transparent, and potentially increase effectiveness. Trialists are encouraged to feed back on the utility of this tool to assist with their trial design and conduct.

### Strengths and limitations

The aim of this study was to identify literature relevant for informing how best to design and implement incentive schemes in clinical trials. We are not aware of any other specific guidance in the literature, and this paper therefore has the potential to inform further developments in this area. We combined theoretical and empirical studies, and structured the review findings to provide maximum transparency and clear guidance.

We conducted a ‘scoping review’ to map existing evidence, and used that to develop initial guidance in a checklist to assist decision-making about incentive design. The development of the checklist represents something of an extension to the usual outputs of a scoping review – although identification of key concepts in a content area is within the remit for a scoping review, the development of practical guidance represents an additional step. We highlight the preliminary nature of our checklist, which is designed to stimulate teams to structure their process when they design incentives, rather than make strong recommendations about the specifics of incentive design.

Our prior knowledge of this area suggested that a conventional systematic review was unlikely to be fruitful because of the lack of primary evidence [4, 5, 60]. Instead, we conducted a scoping review drawing on a range of theoretical and empirical evidence, and developed guidance based on our interpretation of this evidence base. This less restrictive approach allowed us to bring together a wide range of both theoretical and empirical literature from different settings in an informative way to address our study aim. Where possible, we drew on evidence from systematic reviews to ensure that our conclusions were supported by rigorous evidence.

Nevertheless, it is important to be aware of the limitations of this scoping review. Only three databases were searched, and no formal quality assessment was undertaken. Although the focus on systematic reviews and trials would have meant that at least some quality appraisal informed selection for the review, there was no formal linking of the checklist content to the quality of the underlying evidence (although the weight of evidence in terms of number of studies was considered). This could be managed through a fuller review of the literature, or using methods for assessing expert opinions such as a Delphi.

We excluded solely qualitative studies, due to limitations in the resources available for the review and a need to restrict the scope. Qualitative research is also less prevalent within the economic literature which was our focus. Nevertheless, qualitative studies could have a very useful role to play in the development, implementation and evaluation of incentives. They could allow exploration of how incentive schemes are understood by patients and professionals, the potential operation of perverse incentives, and the impact of ethical issues that might be raised by their use [61]. We would certainly encourage users of incentives schemes to embed qualitative process work to explore these issues [62].

Making conclusions based on evidence from settings other than trials requires caution as the effects of incentives may be context specific. There may be justified concerns about generalising results, as the behaviours targeted in a lifestyle behaviour change intervention (such as sustained changes to diet or exercise) are likely to be different from the more episodic and time-limited behaviour required in trials (such as clinic visits and completion of outcomes measures). To minimise this risk we have clearly identified such data in summaries. Given the lack of reporting on the impact of incentive scheme design, it was necessary to draw on this wider literature as the primary evidence within trials is so limited. Researchers are encouraged to report their incentive schemes, or otherwise make them available to assist with future design.

Within the limited empirical evidence specific to the area of trials, much of it examines incentives for questionnaire responses rather than strategies to improve recruitment and retention when participants are required to return to site for follow-up assessments. There was also far more evidence on incentives directed at participants than at recruiters. The literature on pay for performance in healthcare is likely to be most informative in terms of recruiter incentives, as pay-for-performance incentives tend to target providers rather than patients. The evidence on health behaviour change largely examines patient-directed incentives.

There is more evidence on some incentive design issues than others. For example, whilst it is fairly clear that the literature supports the use of immediate rather than delayed incentives, evidence on the most effective incentive structure is sparse.

Finally, the focus of the literature is on increasing rates of recruitment into trials, but it is also important to explore the types of patients recruited. There is increasing concern about processes impacting on patient selection into trials and the impact on external validity, and it will be important to explore the effects of incentives on these selection processes.

### Implications

We have generated guidance for the development of incentives based on both economic theory and empirical evidence, producing an eight-point checklist for scheme designers to follow. This paper highlights just how complex the design of incentive systems can be, and how crucial each design choice is to overall effectiveness. The most appropriate design choice will differ by situation, and we have aimed to provide context-specific advice.

### Next steps

Continued problems with recruitment and retention and the significant sample size requirements of modern trials highlight the need to develop and test innovative incentive strategies alongside other mechanisms such as patient involvement and improved information for participants. Although the evidence suggests that incentives have the potential to improve both recruitment and retention, there is a need for more evidence on both the effectiveness and efficiency of different incentive schemes to ensure that they are a good use of public funds. Such evidence could be produced through embedded studies within a trial [63], which are increasingly supported by funders [64].

We have concluded the section on each design aspect by highlighting the gaps in the current evidence base. Whilst all design issues warrant further research, our scoping review suggests that evidence is most needed on incentives directed towards recruiters, optimal incentive size, and tests of different incentive structures, particularly exploring potential incentive structures for repeat arrangements with recruiters.

### Supplementary information

**Additional file 1:** Source of the papers identified by the searches.

## Data Availability

Not applicable.

## Appendix

### PubMed (June 2016)

1. Accrual*[Title/Abstract]
2. Recruit*[Title/Abstract]
3. Participat*[Title/Abstract]
4. Enlist*[Title/Abstract]
5. Enrol*[Title/Abstract]
6. (#1 or #2 or #3 or #4 or #5)
7. Incent*[Title/Abstract]
8. (#6 and #7)
9. Trial*[Title/Abstract]
10. Random*[Title/Abstract]
11. (#9 or #12)
12. (#8 and #11)

### EconLit (June 2016)

1. Accrual*.mp
2. Recruit*.mp
3. Participat*.mp
4. Enlist*.mp
5. Enrol*.mp
6. incent*.mp
7. 1 or 2 or 3 or 4 or 5
8. 6 and 7
9. Trial*.mp
10. Experiment*.mp
11. Random*.mp
12. 9 or 10 or 11
13. 8 and 12
