# Supplementary material for: Designing and using incentives to support recruitment and retention in clinical trials: a scoping review and a checklist for design
Source: Trials. 2019 Nov 9;20:624. doi: 10.1186/s13063-019-3710-z (PMC6842495; doi:10.1186/s13063-019-3710-z)
Supplement: Supplementary file 1 — Additional file 1: Source of the papers identified by the searches. [file 13063_2019_3710_MOESM1_ESM.docx]

1. Treweek S, Pitkethly M, Cook J, Fraser C, Mitchell E, Sullivan F, et al. Strategies to improve recruitment to randomised trials. Cochrane Database Syst Rev [Internet]. 2018 [cited 2018 Aug 9];(2). Available from: <https://www.cochranelibrary.com/cdsr/doi/10.1002/14651858.MR000013.pub6/abstract>
2. Bower P, Brueton V, Gamble C, Treweek S, Smith CT, Young B, et al. Interventions to improve recruitment and retention in clinical trials: a survey and workshop to assess current practice and future priorities. Trials. 2014 Oct 16;15:399.
3. Raftery J, Bryant J, Powell J, Kerr C, Hawker S. Payment to healthcare professionals for patient recruitment to trials: systematic review and qualitative study. Health Technol Assess [Internet]. 2008;12(10). Available from: <https://www.journalslibrary.nihr.ac.uk/hta/hta12100#/abstract>
4. Asch D, Troxel A, Stewart W, Sequist T, Jones J, Hirsch A, et al. Effect of Financial Incentives on Lipid Levels. JAMA. 314(18):1926–35.
5. Cahill K, Hartmann-Boyce J, Perera R. Incentives for smoking cessation. Cochrane Database Syst Rev. 2015 May 18;(5):CD004307.
6. Paul-Ebhohimhen V, Avenell A. Systematic review of the use of financial incentives in treatments for obesity and overweight. Obes Rev Off J Int Assoc Study Obes. 2008 Jul;9(4):355–67.
7. Kane RL, Johnson PE, Town RJ, Butler M. Economic Incentives for Preventive Care: Summary [Internet]. Agency for Healthcare Research and Quality (US); 2004 [cited 2018 Mar 23]. Available from: <https://www.ncbi.nlm.nih.gov/books/NBK11845/>
8. Pit SW, Vo T, Pyakurel S. The effectiveness of recruitment strategies on general practitioner’s survey response rates - a systematic review. BMC Med Res Methodol. 2014 Jun 6;14:76.
9. Lutge EE, Wiysonge CS, Knight SE, Sinclair D, Volmink J. Incentives and enablers to improve adherence in tuberculosis. Cochrane Database Syst Rev. 2015 Sep 3;(9):CD007952.
10. Halpern SD, Karlawish JHT, Casarett D, Berlin JA, Asch DA. Empirical assessment of whether moderate payments are undue or unjust inducements for participation in clinical trials. Arch Intern Med. 2004 Apr 12;164(7):801–3.
11. Jennings CG, MacDonald TM, Wei L, Brown MJ, McConnachie L, Mackenzie IS. Does offering an incentive payment improve recruitment to clinical trials and increase the proportion of socially deprived and elderly participants? Trials. 2015 Mar 7;16:80.
12. Rendell JM, Merritt RK, Geddes J. Incentives and disincentives to participation by clinicians in randomised controlled trials. Cochrane Database Syst Rev [Internet]. 2007 [cited 2018 Aug

FROM ORCCA

1. David MC, Ware RS. Meta-analysis of randomized controlled trials supports the use of incentives for inducing response to electronic health surveys. J Clin Epidemiol. 2014 Nov;67(11):1210–21.
